# Refractive Index Sensing Based on Fano Resonances of a Bus Metal–Insulator–Metal Waveguide with D-Shaped Resonant Cavity

**DOI:** 10.3390/mi17080946

**Published:** 2026-08-08

**Authors:** Yaner Qiu, Xuyan Chang, Dongqing Zhao, Zhidong Zhang

**Affiliations:** 1 Department of Physics and Electronic Information Engineering, Lyuliang University, Lvliang 033001, China; 2 Lvliang City Key Laboratory of Quantum Precision Measurement, Lyuliang University, Lvliang 033001, China; 3 Key Laboratory of Instrumentation Science & Dynamic Measurement of Ministry of Education, North University of China, Taiyuan 030051, China

**Keywords:** surface plasmon polaritons, metal–insulator–metal (MIM) waveguide, D-shaped resonance cavity, Fano resonance, refractive index sensor

## Abstract

A plasmonic waveguide coupling cavity structure is proposed, consisting of a bus metal–insulator–metal (MIM) waveguide-coupled single/double D-shaped resonant cavity. The transmission properties, magnetic field distribution, and refractive index sensing performance are simulated using the finite element method (FEM). A clear multi-Fano resonance phenomenon is observed in the transmission spectra. The Fano resonances arise from the interaction between the discrete states of the D-shaped resonant cavity and the continuum state of the bus MIM waveguide. The influence of various structural parameters on the Fano resonance is investigated. Furthermore, the refractive index sensing properties based on the Fano resonance are studied by varying the refractive index of the insulator layer. Maximum sensitivities of 1130 nm/RIU and 1150 nm/RIU are achieved at the fourth Fano resonance dip for the single and double D-shaped resonator cavities, respectively. And a maximum figure of merit of 171 is achieved by the single D-shaped cavity structure. This research can provide new avenues for the design of high-sensitivity sensors as well as novel on-chip sensing structures.

## 1. Introduction

Surface plasmon polaritons (SPPs) are a collective oscillation mode excited by the interaction between incident light and free electrons on the surface of noble metal nanostructures [1,2]. It can tightly confine electromagnetic energy at the metal–dielectric interface, with an exponential decay in the direction perpendicular to the interface, thereby exhibiting significant subwavelength localization and near-field enhancement effects [3,4]. So it is able to overcome the conventional optical diffraction limit [5]. SPPs enable effective manipulation of optical fields at the nanoscale, making them an ideal physical platform for constructing ultra-compact integrated optoelectronic devices such as sensors, modulators, and detectors [6,7,8].

The metal–insulator–metal (MIM) waveguide coupling resonant cavity structure provides a broad platform for developing high-performance photonic devices, including ultra-compact filters, high-sensitivity biosensors, and optical isolators [9,10,11]. In recent years, Fano resonance, a weak interaction in quantum systems, manifests in optical spectra as a highly characteristic asymmetric line shape and has been observed in this coupled system [12,13,14,15]. This phenomenon exhibits a higher quality factor (Q-factor) and steeper phase variation, meaning it is extremely sensitive to minute perturbations in the surrounding dielectric environment or slight changes in structural parameters [16,17]. This outstanding spectral sensitivity endows Fano resonance with tremendous application potential in biochemical sensing [18,19,20,21]. Therefore, how to design novel MIM waveguide-coupled resonator topologies to excite high-contrast Fano resonances and optimize their sensing performance, while maintaining structural compactness and fabrication feasibility, has become a key scientific issue and research hotspot in the field of nanophotonics [22]. To optimize sensing performance, various resonator structures have been proposed, including double rectangular cavities [13], cross-shaped cavities, and X-shaped cavities [23,24,25]. Although these designs achieve commendable sensitivity, they often suffer from complex fabrication processes. Among various types of resonator-coupled MIM waveguide structures, the D-shaped resonant cavity offers advantages such as ease of fabrication, strong mode tunability, and significant field localization, making it an ideal candidate for constructing high-performance plasmonic sensors. Compared with traditional circular or rectangular cavities, the D-shaped resonant cavity maintains strong light confinement, which is beneficial for generating multimode interference and Fano resonances. For example, Liu et al. (2020) demonstrated a Fano resonance sensor based on a single D-shaped cavity coupled with a bus waveguide containing a silver–air–silver barrier [26]. However, introducing such physical barriers inevitably increases nanofabrication complexity. Furthermore, most previous work has focused on single cavities, with limited exploration of multimode interference and mode splitting in dual-cavity systems.

Therefore, this paper proposes a structurally simple and baffle-free plasmonic sensing system consisting of a bus MIM waveguide coupled with a D-shaped resonant cavity. The transmission spectra and normalized magnetic field distributions of the system are simulated and analyzed using the finite element method (FEM). By analyzing the steady-state magnetic field distributions at each resonant wavelength, the generation mechanisms of different Fano resonance modes are explored. Furthermore, the effects of geometric parameter variations in the D-shaped resonant cavity on Fano resonances are discussed, and the sensing performance of the structure is systematically investigated based on these resonance dips.

## 2. Structure Model and Analytical Method

**Figure 1:**
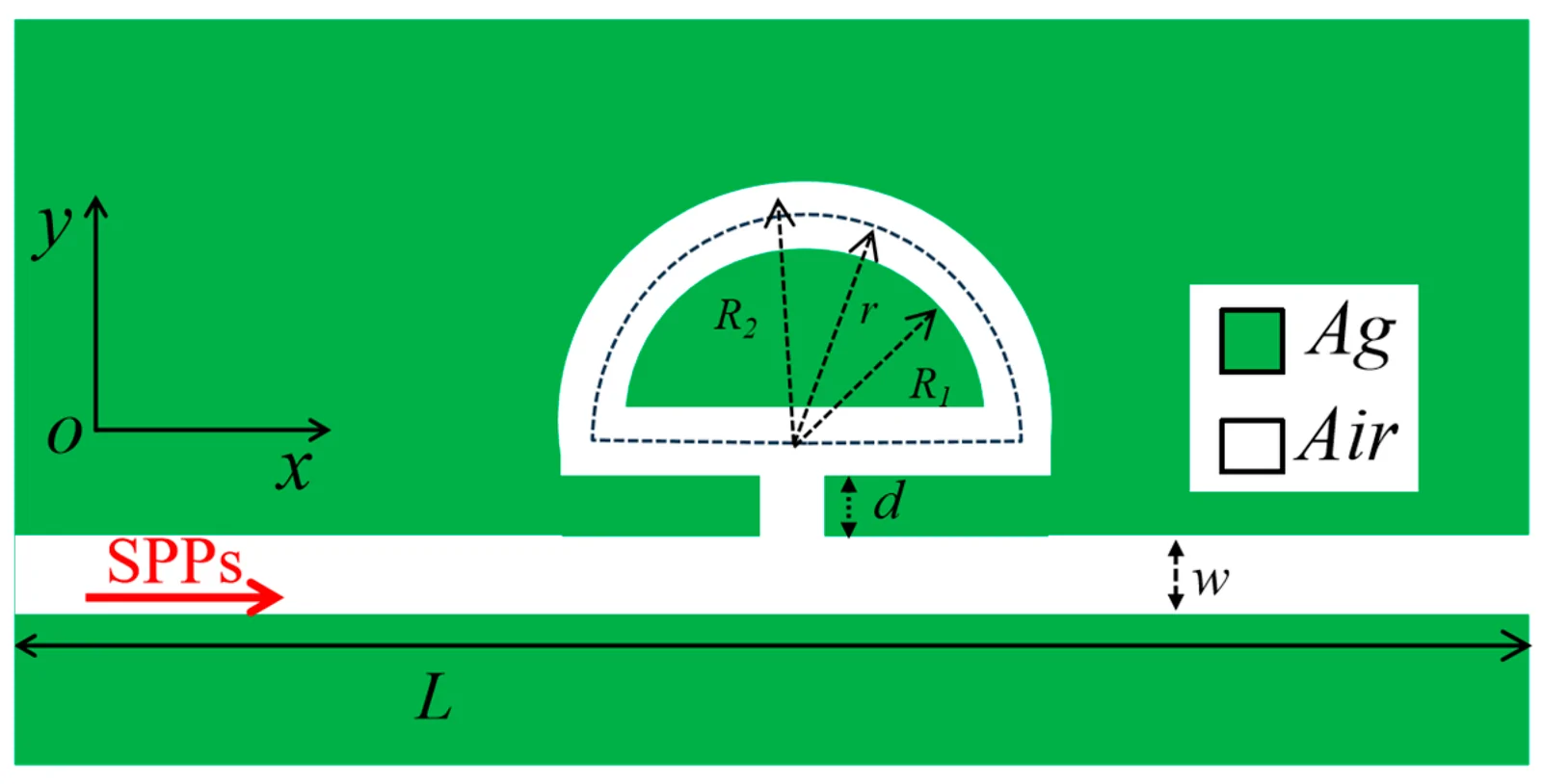
Two-dimensional schematic for the bus MIM waveguide-coupled D-shaped resonant cavity.

The 2D schematic of the proposed structure (as shown in Figure 1) consists of a bus MIM waveguide coupled with a D-shaped resonant cavity. In Figure 1, the green and white regions represent Ag (*ε_m_*) and air (*ε_s_*), respectively. The width of both the MIM waveguide and the D-shaped resonant cavity is fixed at *w* = 50 nm, which can only support the fundamental transverse magnetic (TM_0_) mode in the MIM waveguide [23,24]. The length of the bus MIM waveguide is denoted by *L*. The distance between the D-shaped resonant cavity and the bus MIM waveguide is given by *d*. The inner and outer radii of the D-shaped resonant cavity are denoted as *R*_1_ and *R*_2_, respectively, with the center radius of the ring cavity calculated as *r* = (*R*_1_ + *R*_2_)/2.

The frequency-dependent complex relative permittivity *ε*(*ϖ*) of silver is characterized by the modified Debye dispersion mode [27,28,29] as follows:

(1) $$\varepsilon (\omega )=\varepsilon_{\infty }+\frac{\left(\varepsilon_{s}-\varepsilon_{\infty }\right)}{1+i\omega \tau }+\frac{\sigma }{i\omega \varepsilon_{0}},$$

where *ε*_∞_ = 3.8344 is the infinite frequency permittivity, *ε_s_* = −9530.5 represents the static permittivity, *σ* = 1.1486 × 10^7^ S/m is the conductivity, and *τ* = 7.35 × 10^−15^ s is the relaxation time.

The transmission properties of the bus MIM waveguide coupled with a D-shaped resonant cavity are investigated by using the FEM with perfectly matched layer absorbing boundary conditions, implemented in the COMSOL Multiphysics software (version 6.3). The transmittance is defined as *T* = |*S*_21_|^2^, where *S*_21_ is the transmittance of the MIM waveguide [13].

To fundamentally elucidate the broadband transmission properties of the proposed plasmonic structure, a theoretical analytical model based on the coupled-mode theory (CMT) is established. The Fano resonance system consists of a broadband continuous state and a narrowband discrete state. Surface plasmon polaritons (SPPs) propagate along the straight waveguide serving as the continuous state, with an incident wave amplitude of *S_in_* and a transmitted wave amplitude of *S_out_*. The rectangular gap *d* between the waveguide and the microcavity introduces a structural perturbation. This perturbation does not lead to mode decoupling; instead, it introduces an inherent background transmittance *t*_0_ and a perturbation-induced system phase shift *ϕ_d_* to the entire system [30,31].

For the discrete states within the D-shaped cavity, the coupled SPP energy excites a specific resonance order. For a given resonance order, two dominant modes are defined in the cavity: the high-energy branch corresponding to the short-wavelength region (with amplitude *a*_1_ and resonance frequency *ω*_1_) and the low-energy branch corresponding to the long-wavelength region (with amplitude *a*_2_ and resonance frequency *ω*_2_). Considering energy conservation, the differential equations governing the temporal evolution of the amplitudes for the high-energy and low-energy branches can be expressed as follows:

(2) $$\frac{da_{1}}{dt}\;=\;(j\omega_{1}\;-\;\gamma_{i1}\;-\;\gamma_{c1})a_{1}\;+\;\sqrt{\gamma_{c1}}S_{in}$$

(3) $$\frac{da_{2}}{dt}=(j\omega_{2}-\gamma_{i2}-\gamma_{c2})a_{2}+\sqrt{\gamma_{c2}}S_{in}$$

where $\gamma_{i1}$ and $\gamma_{i2}$ represent the intrinsic loss rates of the high-energy and low-energy branches, respectively, and $\gamma_{c1}$ and $\gamma_{c2}$ denote their coupling loss rates with the bus waveguide. Assuming the input light is a continuous harmonic wave $S_{in}\propto e^{jwt}$, the steady-state amplitudes in the frequency domain are derived as follows:

(4) $$a_{1}\;=\;\frac{\sqrt{\gamma_{c1}}S_{in}}{j(\omega \;-\;\omega_{1})\;+\;\gamma_{i1}\;+\;\gamma_{c1}}$$

(5) $$a_{2}=\frac{\sqrt{\gamma_{c2}}S_{in}}{j(\omega -\omega_{2})+\gamma_{i2}+\gamma_{c2}}$$

The final output field *S_out_* is the coherent superposition of the directly transmitted wave (affected by the waveguide perturbation) and the resonant radiated waves leaking from the D-shaped cavity back into the waveguide. By substituting $a_{1}$ and $a_{2}$ into the output field expression and dividing both sides by *S_in_*, the complex amplitude transmittance *t*(*w*) for this system is obtained:

(6) $$t(\omega )\;=\;\frac{S_{out}}{S_{in}}\;=\;t_{0}e^{j\phi_{d}}\;-\;\frac{\gamma_{c1}e^{j\theta_{1}}}{j(\omega \;-\;\omega_{1})\;+\;\gamma_{i1}\;+\;\gamma_{c1}}\;-\;\frac{\gamma_{c1}e^{j\theta_{2}}}{j(\omega \;-\;\omega_{2})\;+\;\gamma_{i2}\;+\;\gamma_{c2}}$$

where $\theta_{1}$ and $\theta_{2}$ are the additional coupling phases when the high-energy and low-energy branches radiate into the waveguide, respectively. The theoretical light intensity transmittance is determined by *T*(*w*) = |t(ω)|^2^.

## 3. Results and Discussion

**Figure 2:**
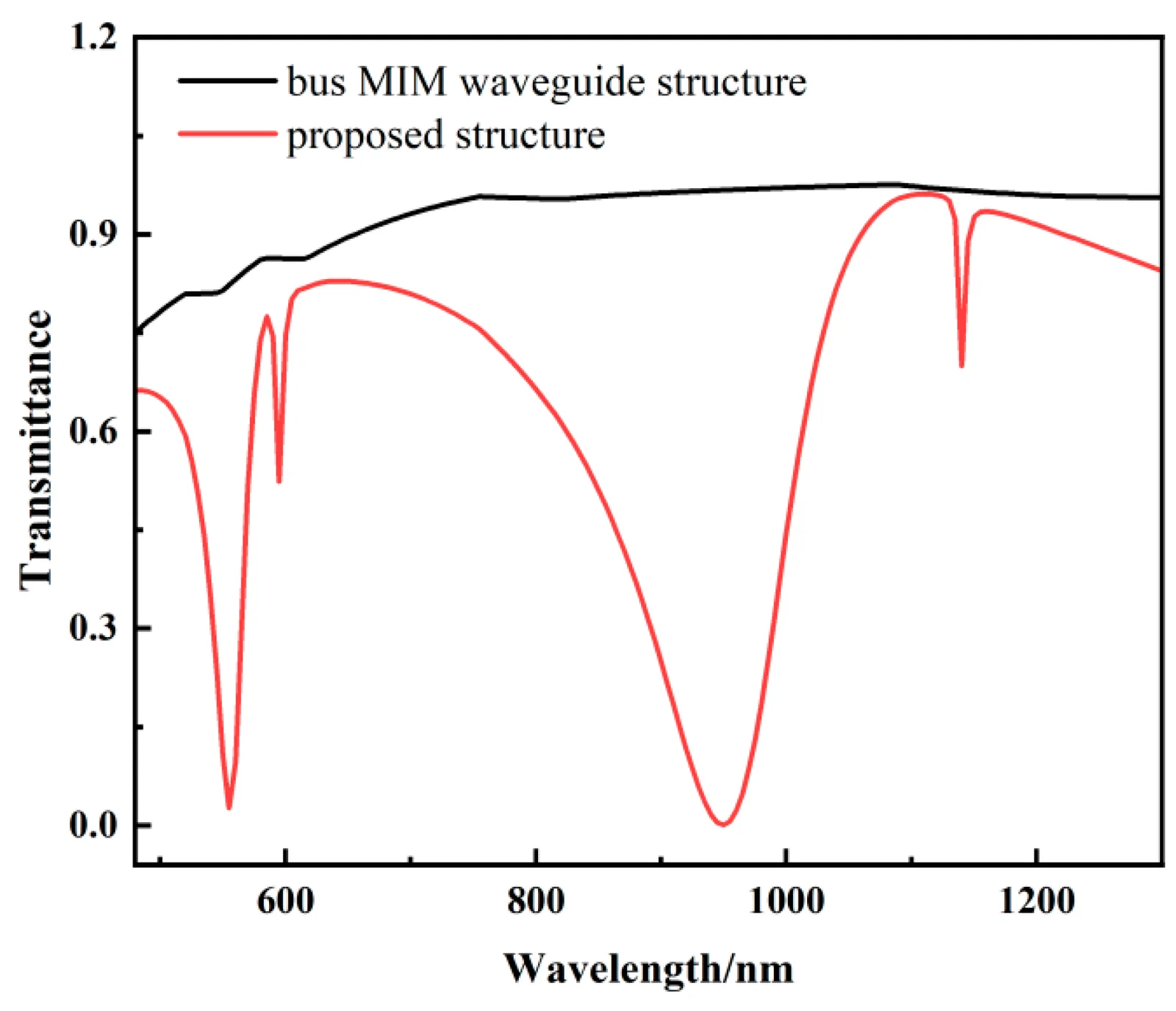
Illustration of the transmission spectra for two configurations: a bus MIM waveguide (black solid line) and the bus waveguide-coupled D-shaped resonant cavity (proposed structure) (red solid line).

Figure 2 illustrates the transmission spectra for two configurations: a single straight waveguide and the straight waveguide joined to a D-shaped resonant cavity (proposed structure), using parameters *d* = 50 nm, *R*_1_ = 130 nm, *R*_2_ = 180 nm, and *r*_2_ = (*R*_1_ + *R*_2_)/2 = 155 nm, *L* = 1200 nm. For the single straight waveguide, a broad spectrum without a resonance peak is observed (black solid line). In contrast, the proposed structure exhibits four distinct transmission dips (red solid line). These resonance dips exhibit distinct Fano resonance line shapes in the transmission spectrum. The transmission dips of the proposed structure are at wavelengths *λ*_1_ = 1140 nm, *λ*_2_ = 950 nm, *λ*_3_ = 595 nm, and *λ*_4_ = 555 nm.

**Figure 3:**
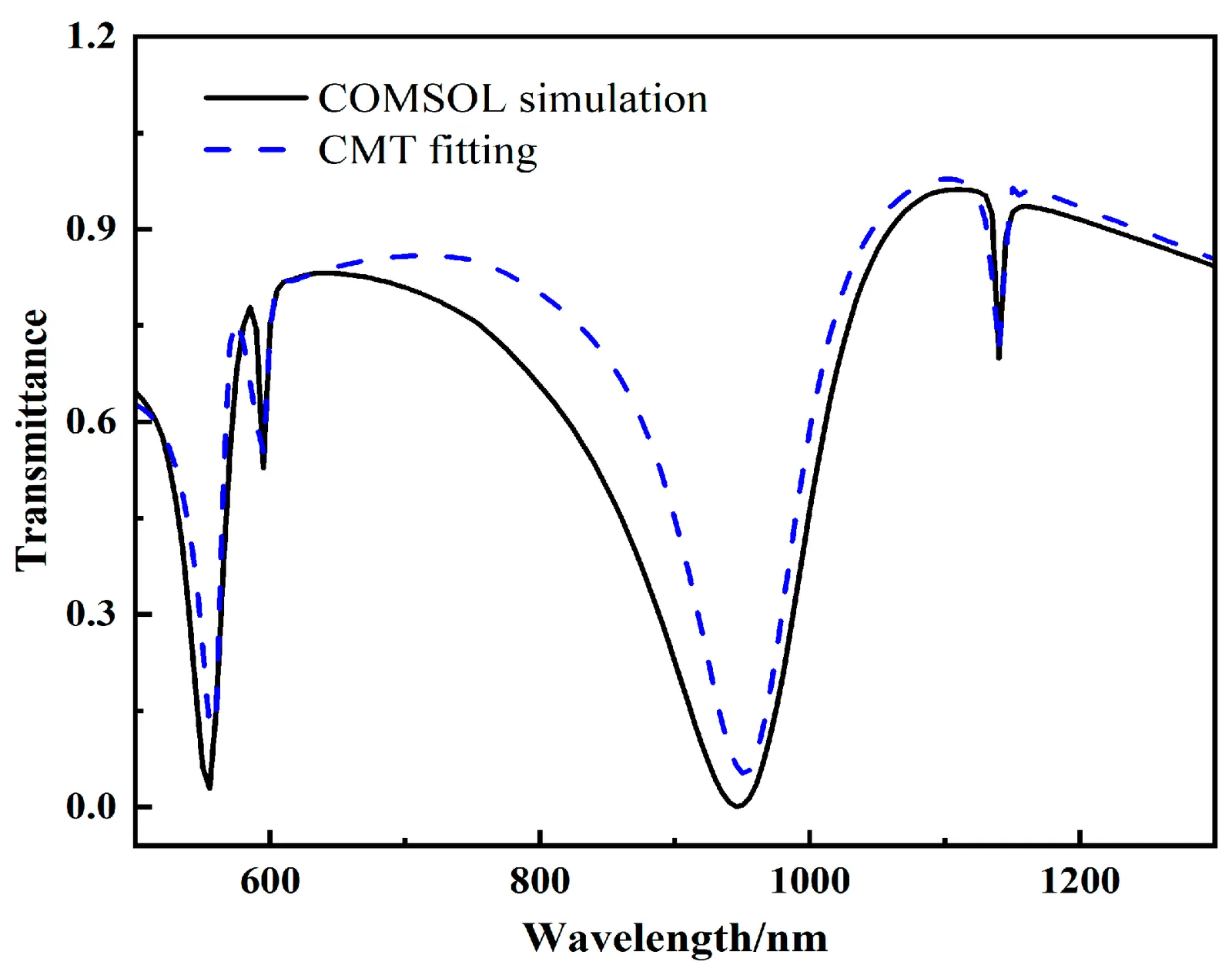
Comparison of the transmission spectra obtained from numerical FEM simulations (black solid line) and analytical CMT fitting (blue solid line) for the proposed structure.

To verify the physical mechanism of the multiple Fano resonances, the analytical CMT model is fitted to the FEM simulation results. Since the D-shaped cavity simultaneously excites different order modes of the high- and low-energy branches within the 500–1300 nm range, direct global fitting is prone to overfitting. Therefore, the broad spectrum is divided into short-wavelength and long-wavelength subsystems, and the two-mode CMT formula is applied separately to extract the parameters. As shown in Figure 3, the transmission spectrum obtained by the piecewise fitting matches well with the FEM data, indicating the good applicability of this method.

**Figure 4:**
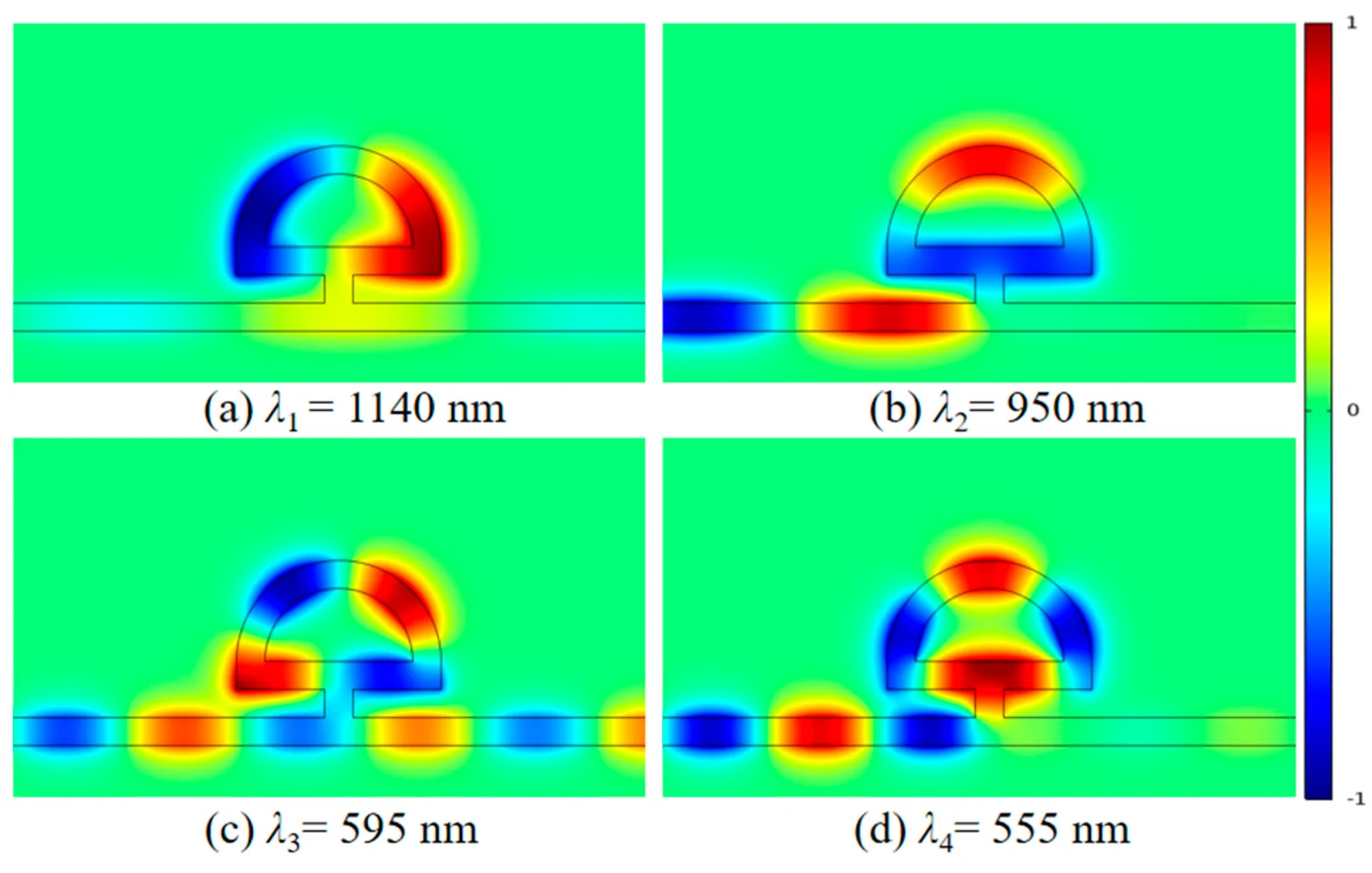
Contour profiles of the normalized *Hz* field distributions of the bus MIM waveguide D-shaped resonant cavity: (**a**) *λ*_1_ = 1140 nm, (**b**) *λ*_2_ = 950 nm, (**c**) *λ*_3_ = 595 nm, and (**d**) *λ*_4_ = 555 nm.

To elucidate the physical mechanism of Fano resonance in a bus waveguide-coupled D-shaped resonant cavity, we calculated the normalized magnetic field distributions at the four Fano resonance dips (*λ*_4_ = 555 nm, *λ*_3_ = 595 nm, *λ*_2_ = 950 nm, and *λ*_1_ = 1140 nm), as shown in Figure 4a–d. Based on the mode order and field distribution characteristics, these resonances can be classified into two categories: short-wavelength resonances (*λ*_3_ = 595 nm, and *λ*_4_ = 555 nm) correspond to second-order modes, while long-wavelength resonances (*λ*_1_ = 1140 nm, and *λ*_2_ = 950 nm) correspond to first-order modes. In the second-order modes, due to specific field distribution characteristics, antibonding states (high-energy states) and bonding states (low-energy states) are formed. For the antibonding state at *λ*_4_ = 555 nm, the electric field energy is highly concentrated in the two “shoulder” regions of the D-shaped resonant cavity, with a distinct low-electric-field region formed at the junction between the D-shaped resonant cavity and the straight waveguide. This strong localization results in most of the electromagnetic energy being confined within the D-shaped resonant cavity, with a small mode volume and significant local field enhancement. In contrast, for the bonding state at *λ*_3_ = 595 nm, the electric field energy is primarily distributed at the junction between the D-shaped resonant cavity and the straight waveguide, one corner, and the opposite curved region. Consequently, part of the energy is confined within the cavity, while the rest can be transmitted to the other end of the straight waveguide. In the first-order modes, bonding and antibonding states are also distinguished. For the antibonding state at *λ*_2_ = 950 nm, the electric field energy is mainly concentrated in the horizontal part of the D-shaped resonant cavity, with the electromagnetic energy almost completely confined within the D-shaped resonant cavity. For the bonding state at *λ*_1_ = 1140 nm, the electric field energy is mostly concentrated in the two corners of the resonant cavity and their adjacent regions. Due to the larger mode volume of the electromagnetic energy, part of the energy is transmitted to the other end of the waveguide, while another part remains confined within the D-shaped resonant cavity. Therefore, the longer-wavelength resonances (*λ*_1_ = 1140 nm, and *λ*_2_ = 950 nm) correspond to lower-order resonant modes with larger mode volumes, where energy is primarily localized inside the cavity and in the near-field coupling region with the waveguide. In contrast, the shorter-wavelength resonances (*λ*_3_ = 595 nm, and *λ*_4_ = 555 nm) correspond to higher-order, small-mode-volume modes, with more nodes or antinodes appearing in the field distribution. The depth, width, and shift of dips in the transmission spectrum depend on the field distribution of the resonant modes within the cavity and their degree of overlap with the evanescent field of the waveguide. Long-wavelength modes, characterized by large mode volumes and strong coupling efficiency, are suitable for sensing and tuning applications. Short-wavelength modes, with strong field localization, are suitable for nonlinear and enhanced spectroscopy applications.

**Figure 5:**
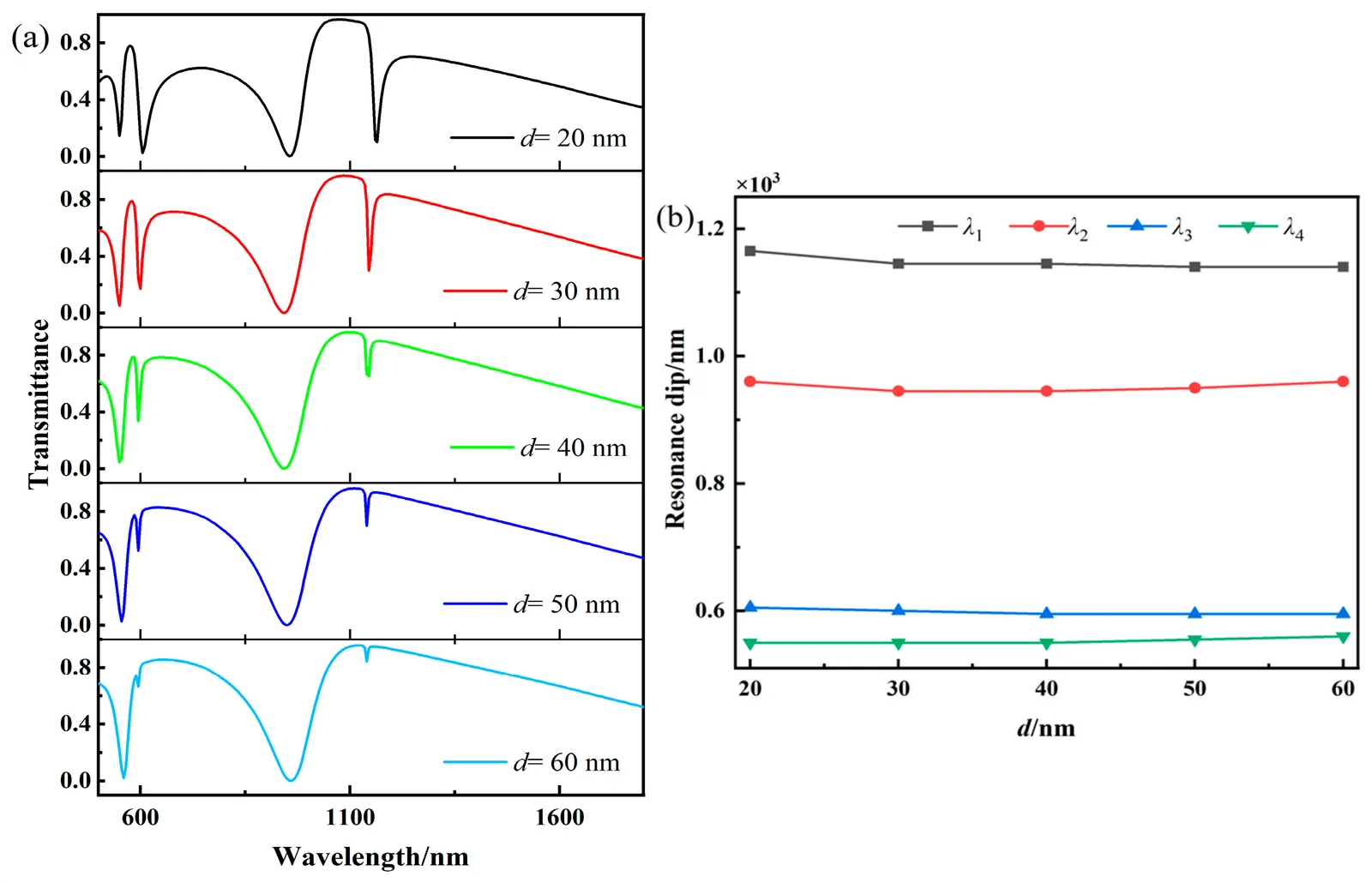
(**a**) Transmission spectra of the bus MIM waveguide-coupled D-shaped resonant cavity with changing *d*; (**b**) the shift of the resonance dip with changing *d*.

To investigate the effect of the distance between the D-shaped resonator and the waveguide on the transmission spectrum, we calculated the transmission spectra by varying the value of *d* from 20 nm to 60 nm in increments of 10 nm, while keeping *r* at 155 nm. The results are shown in Figure 5a. We found that the transmittance of the first Fano resonance dip gradually increases with increasing *d*, and the dip becomes shallower. The second Fano resonance dip broadens in shape, but its transmittance does not change significantly. With increasing *d*, the transmittance at the third Fano resonance dip increases, corresponding to a shallower transmission dip. The transmittance of the fourth Fano resonance dip decreases with increasing *d*, i.e., the dip becomes deeper. This indicates that the first and third Fano resonance dips are mainly influenced by *d*, whereas the second and fourth Fano resonance dips are less affected by *d*. As *d* increases, the four Fano resonance dips do not shift significantly. Therefore, the increase in *d* mainly affects the coupling efficiency rather than the resonance condition itself. The resonance wavelength is primarily determined by the geometric dimensions and refractive index of the D-shaped resonator, while *d* mainly governs the energy coupling between the waveguide and the resonator.

**Figure 6:**
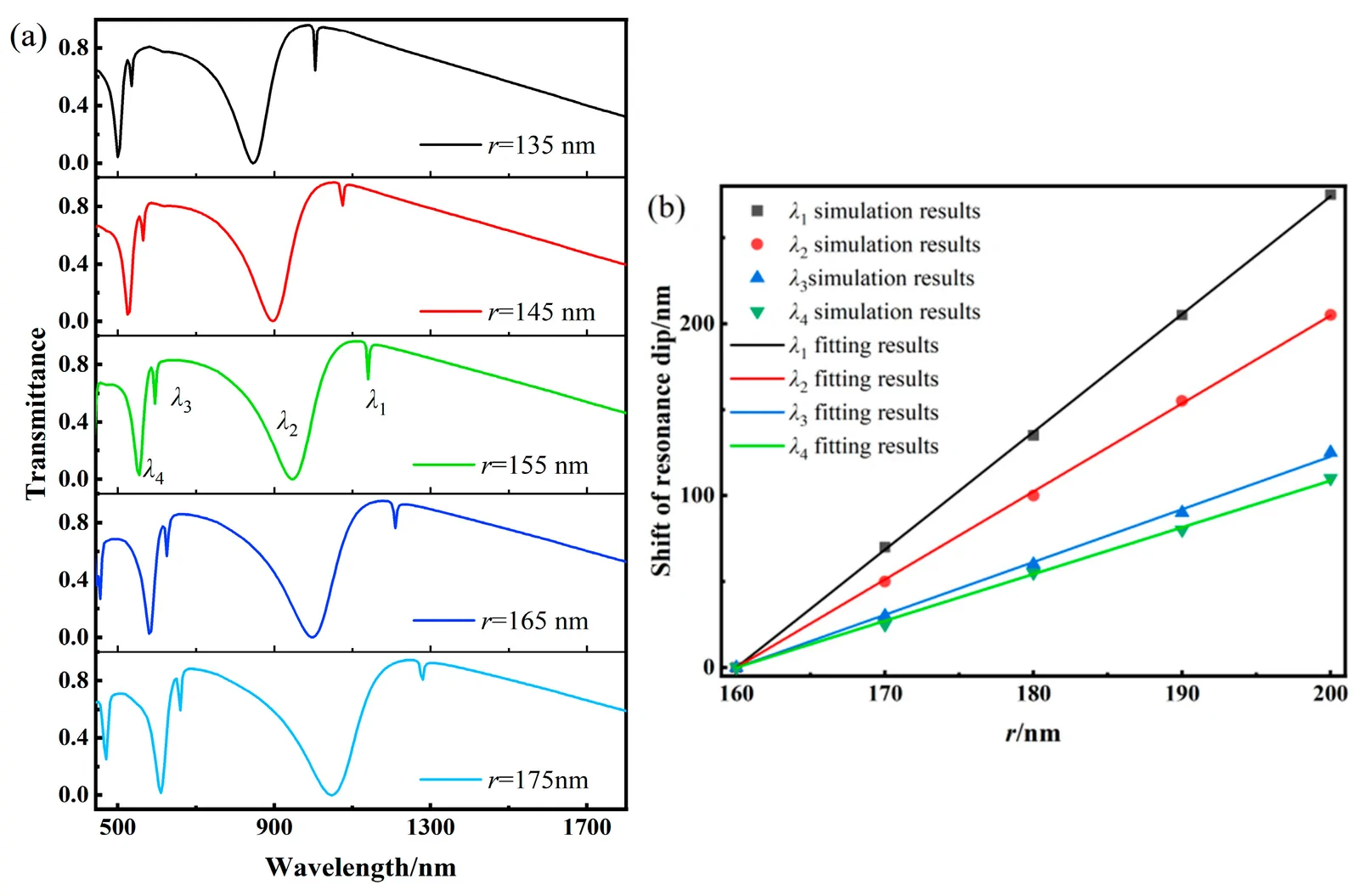
(**a**) Transmission spectra of the bus MIM waveguide coupled D-shaped resonant cavity with changing *r*; (**b**) the shift of Fano resonance dip with changing *r*.

Figure 6a shows the transmission spectra of *r* from 135 nm to 175 nm in increments of 10 nm. As shown in Figure 6b, we found that all four Fano resonance dips exhibit a clear red shift as *r* increases. This is because the effective optical path length increases with the increase in *r*, which causes a red shift. This phenomenon indicates that the Fano resonance dip can be effectively tuned by adjusting the geometric dimensions of the D-shaped resonator cavity. For the Fano resonance dips in the short-wavelength region (e.g., 500–800 nm), the transmittance gradually decreases as *r* increases, i.e., the resonance dips become deeper. This suggests that the coupling is enhanced, and energy is more effectively confined within the resonator. For the resonance dips in the long-wavelength region (e.g., 1000–1450 nm), the transmittance slightly increases as *r* increases, and the resonance dips become shallower, indicating that the coupling strength weakens and some modes become more sensitive to external radiation or losses. According to coupled-mode theory (CMT), the extinction depth of a Fano dip is governed by the ratio of the coupling decay rate (*γ_c_*) to the intrinsic decay rate (*γ_i_*). For the higher-order mode, the system initially resides in the over-coupled regime due to its strongly localized optical field (*γ_c_* > *γ_i_*). As the structural parameter *r* varies, the ratio (*γ_c_*/*γ_i_*) gradually approaches unity, leading to a notable deepening of the corresponding resonance dip. In contrast, for the lower-order mode, the system starts near the critical-coupling point. The same variation in *r* causes (*γ_c_*/*γ_i_*) to gradually deviate from unity, thus deteriorating the critical-coupling condition and resulting in a shallower resonance dip instead. As *r* increases, the full widths at half maximum (*FWHM*) of the second and fourth Fano resonance dips gradually increase, indicating that the radiation loss or internal loss of the resonator increases, and the Q-factor decreases. This may be attributed to changes in the curvature of the D-shaped resonator, which alter the mode volume and radiation efficiency.

**Figure 7:**
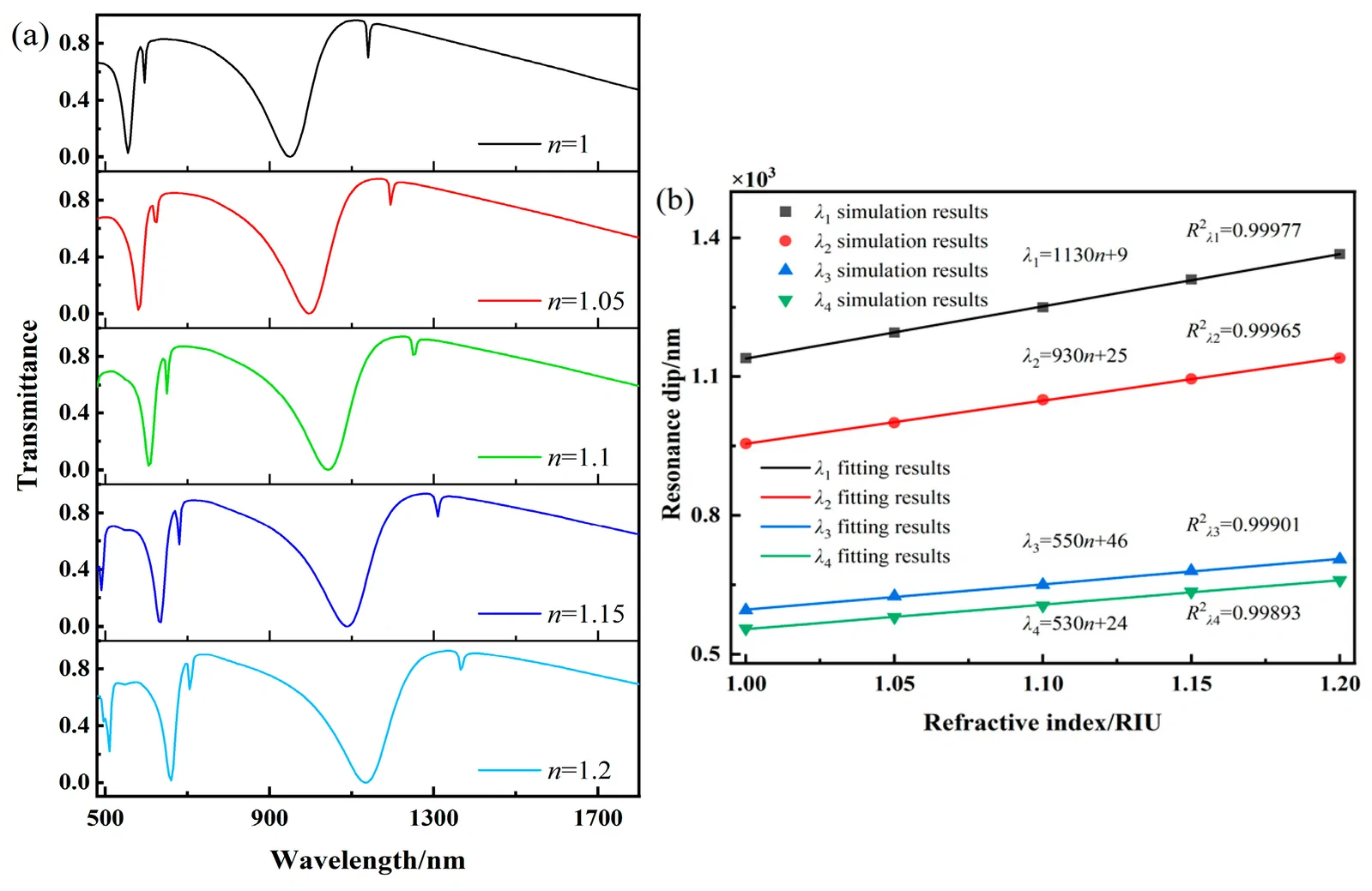
(**a**) Transmission spectra of the bus MIM waveguide-coupled D-shaped resonant cavity with changing *n*; (**b**) the shift of the Fano resonance dips as a function of the refractive index change *n*.

Figure 7 presents the transmission spectra of the medium refractive index *n* in both the waveguide and the cavity varying from 1.0 to 1.2 in increments of 0.05. As *n* gradually increases, the effective optical path length of the resonator becomes longer, which results in a significant red shift of the Fano resonance dips. These Fano resonance dips exhibit pronounced shifts with variations in *n*, indicating that the D-shaped resonator structure possesses excellent refractive index sensing characteristics. This structure is suitable for high-sensitivity biological or chemical sensors. Furthermore, as *n* increases, the first Fano resonance dip gradually becomes shallower, while the other three Fano resonance dips show no significant change. This suggests that the coupling between the bus waveguide and the D-shaped resonator is strong, with energy being effectively confined within the D-shaped resonant cavity. To further quantitatively evaluate the sensitivity characteristics of the refractive index sensor based on the Fano resonance, the sensitivity *S* is used to characterize the sensitivity. The sensitivity (*S*) can be represented as *S* = δ_*λ*_/δ_*n*_, where δ_*λ*_ is the change in the Fano resonance dip, and δ_*n*_ is the change in the environmental refractive index. The refractive index sensitivities of the four Fano resonance dips are *S_λ_*_1_ = 1130 nm/RIU, *S_λ_*_2_ = 950 nm/RIU, *S_λ_*_3_ = 560 nm/RIU, and *S_λ_*_4_ = 500 nm/RIU, respectively. The figure of merit (*FOM*) can be expressed as *FOM* = *S*/*FWHM*. *FWHM* is the full width at half maximum of the resonance dip. The *FOM* of the four Fano resonance dips are *FOM_λ_*_1_ = 171.49 RIU^−1^, *FOM_λ_*_2_ = 6.40 RIU^−1^, *FOM_λ_*_3_ = 80.27 RIU^−1^, and *FOM_λ_*_4_ = 16.73 RIU^−1^.

Subsequently, we compare its sensing performance with several typical MIM waveguide sensors in Table 1. The results indicate that our proposed sensor possesses a competitive sensitivity while maintaining an exceptionally high *FOM* and Q-factor.

**Figure 8:**
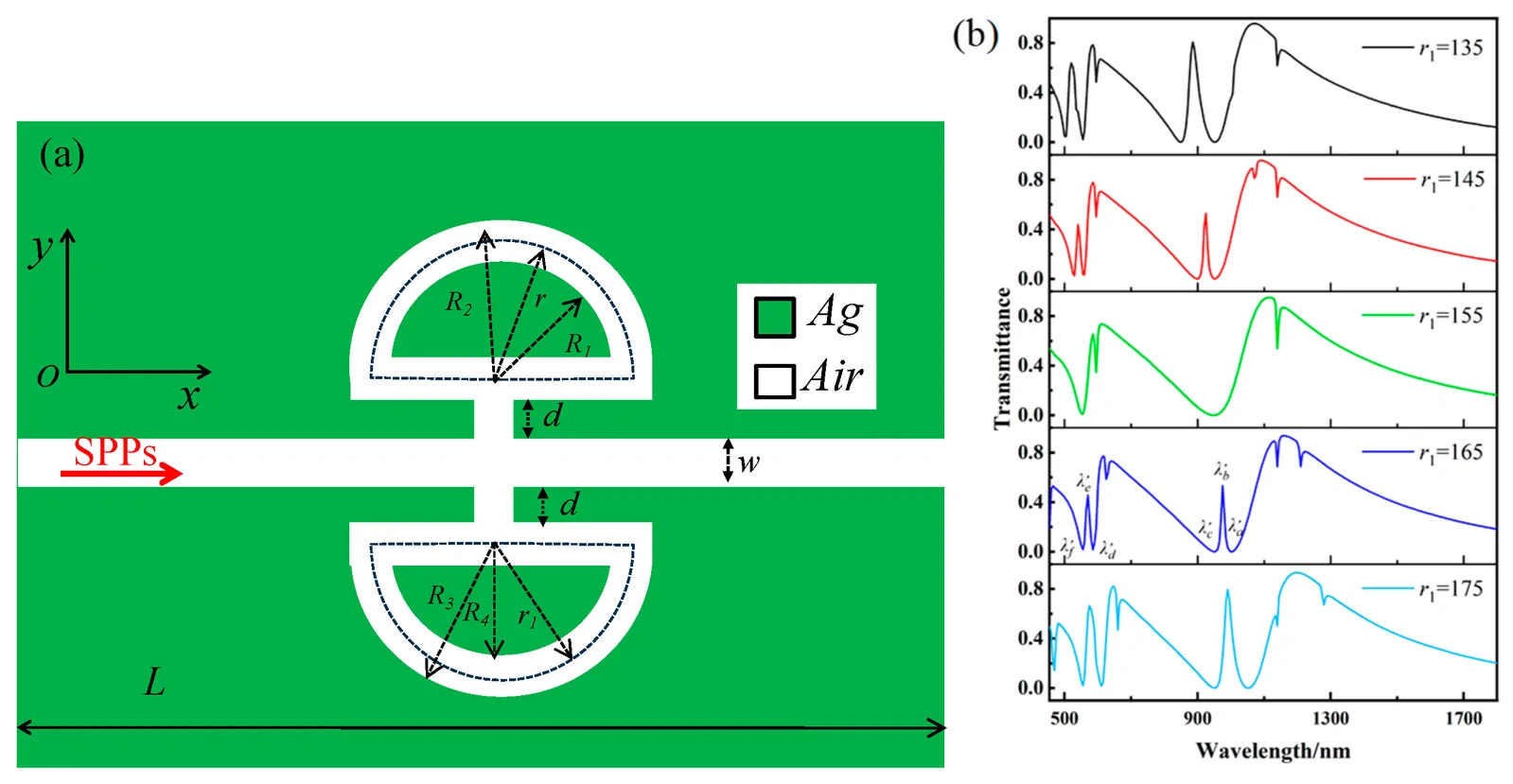
(**a**) Two-dimensional schematic for the bus MIM waveguide-coupled double D-shaped resonant cavity; (**b**) transmission spectra of the bus MIM waveguide-coupled double D-shaped resonant cavity with changing *r*_1_.

To investigate the modulation mechanism of structural parameters on the transmission characteristics of the straight waveguide coupled with double D-shaped resonators (as shown in Figure 8a), we fixed the geometric parameters of the upper D-shaped resonator and the straight waveguide (central radius *r*_1_ = 155 nm and coupling distance *d* = 30 nm) and simulated the evolution of the system’s transmission spectrum as the outer radius *r*_1_ of the lower D-shaped resonator gradually increased from *r*_1_ = 135 nm to *r*_1_ = 175 nm, as shown in Figure 8b.

As can be seen from the figure, the coupled structure excites multiple distinct Fano resonance peaks and dips in the visible to near-infrared region (approximately 400 nm–1600 nm). This asymmetric line shape originates from the coherent interference between the continuously propagating SPP modes in the straight waveguide and the discrete localized modes in the D-shaped resonators. The introduction of the double D-shaped resonant cavity structure enables the system to support richer resonance modes compared to a single cavity, exhibiting complex multimode interference characteristics. As the radius *r*_1_ of the lower resonator increases, the main resonance dips in the transmission spectrum show a significant red-shift trend. This is because the increase in radius leads to a longer perimeter of the D-shaped resonant cavity, thereby increasing the effective optical path length for SPP propagation within the cavity. According to the standing wave resonance condition, the resonance wavelength consequently shifts towards the long-wavelength direction.

It is worth noting that as *r*_1_ gradually increases from *r*_1_ = 135 nm to *r*_1_ = 175 nm, the system undergoes an evolution process from “asymmetric” to “completely symmetric” and back to “asymmetric”. Particularly at the completely symmetric point (*r*_1_ = 155 nm), the upper and lower D-shaped cavities have identical geometric dimensions and thus the same eigen-resonance frequencies. Under this condition, the two resonators are strongly coupled via the evanescent field in the intermediate waveguide region, leading to mode splitting of the originally degenerate energy levels. This phenomenon manifests in the transmission spectrum as the splitting of a single resonance dip into two independent deep dips, corresponding to the in-phase oscillation (bonding state/low-energy state/longer wavelength) and out-of-phase oscillation (antibonding state/high-energy state/shorter wavelength) of the electromagnetic fields in the upper and lower cavities, respectively. This mode modulation mechanism based on symmetry breaking and restoration provides a theoretical basis for the design of multi-channel filters and high-sensitivity sensors.

**Figure 9:**
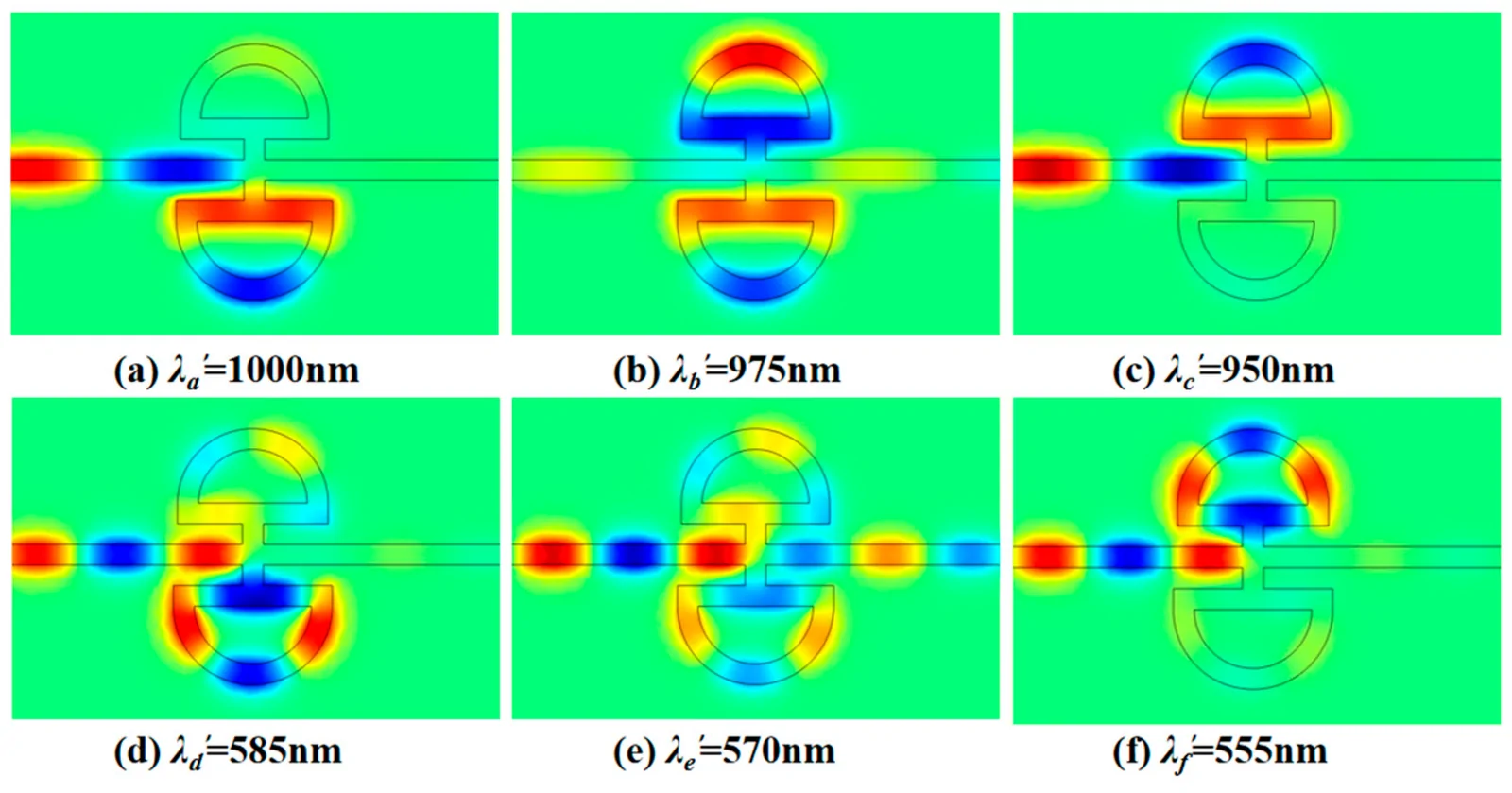
Contour profiles of the normalized *Hz* field distributions of the bus MIM waveguide double D-shaped resonant cavity: (**a**) *λ′_a_* = 1000 nm, (**b**) *λ′_b_* = 975 nm, (**c**) *λ′_c_* = = 950 nm, (**d**) *λ′_d_* = = 585 nm, (**e**) *λ′_e_* = 570 nm, and (**f**) *λ′_f_* = 555 nm.

To deeply reveal the physical mechanism underlying the Fano resonance and transmission enhancement in this structure, we calculated the normalized magnetic field distributions at four characteristic wavelengths in the transmission spectrum under the structural parameter *r*_1_ = 165 nm (as shown in Figure 9). The analysis indicates that the resonance characteristics primarily originate from the coherent interference between the continuously propagating SPP modes in the bus MIM waveguide and the discrete localized modes within the dual D-shaped resonators. Meanwhile, due to the asymmetric configuration of the system (*r* < *r*_1_), the coupling effects between the upper and lower cavities further introduce complex mode hybridization behavior. Specifically, at the transmission dips of the first-order mode (*λ′_a_* = 1000 nm and *λ′_c_* = 950 nm, as shown in Figure 9a,c) and those of the second-order mode (*λ′_d_* = 585 nm and *λ′_f_* = 555 nm, as shown in Figure 9d,f), the magnetic field distributions exhibit a clear wavelength dependence: under short-wavelength excitation, the normalized magnetic field is mainly localized in the D-shaped resonator below the waveguide, whereas under long-wavelength excitation, the field is predominantly concentrated in the resonator above the waveguide. At the transmission minima (*λ′_b_* = 975 nm and *λ′_e_* = 570 nm, as shown in Figure 9b,e), the electromagnetic energy is simultaneously confined within both the upper and lower D-shaped resonators. Notably, for both the first-order and second-order resonance modes, the spatial distribution of the normalized magnetic field within the cavities exhibits an antisymmetric pattern with respect to the waveguide. This antisymmetric distribution enables partial energy to couple into the waveguide, thereby giving rise to a transmission peak in the spectrum.

**Figure 10:**
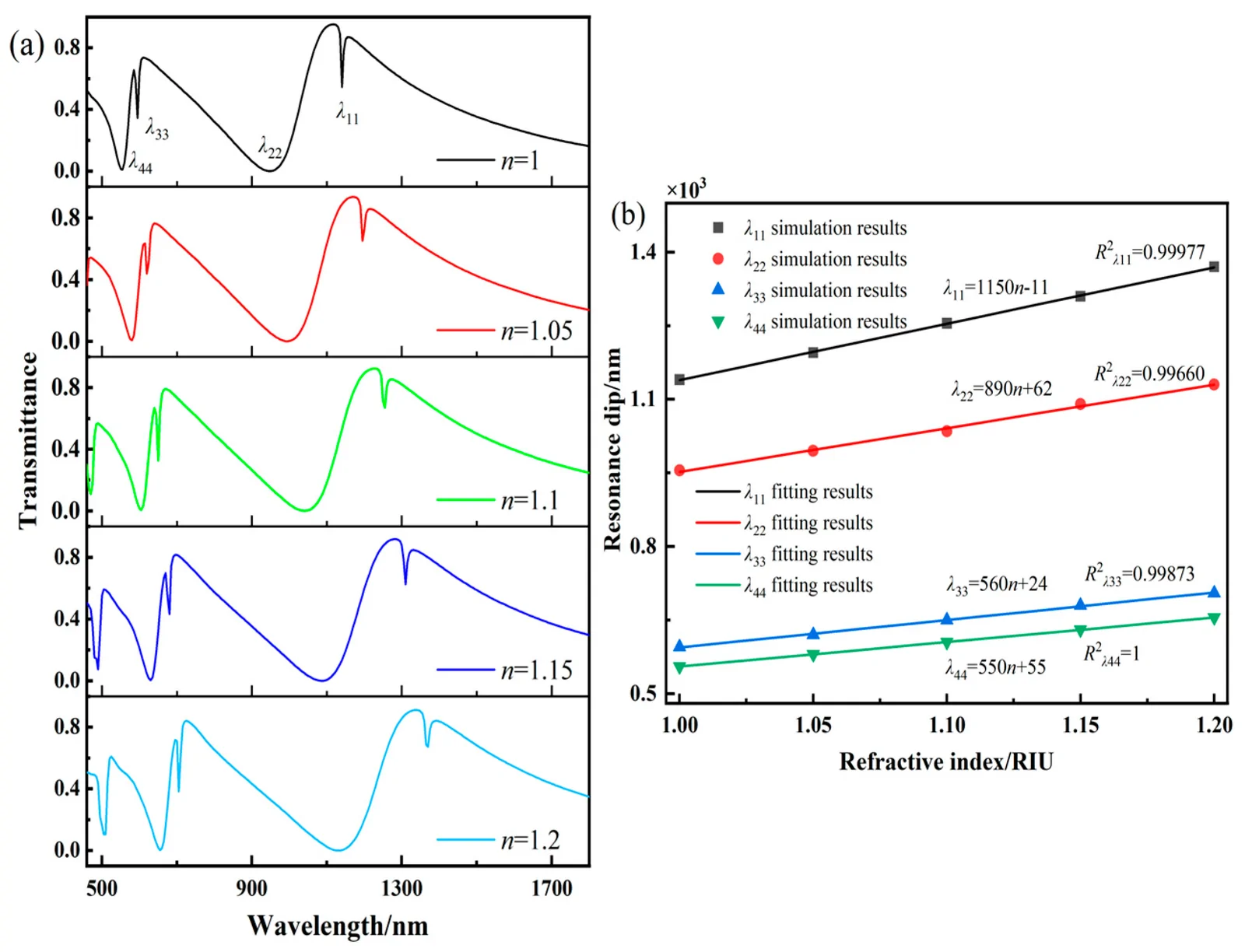
(**a**) Transmission spectra of the bus MIM waveguide-coupled double D-shaped resonant cavity with changing *n*; (**b**) the shift of the Fano resonance dips as a function of the refractive index change *n*.

Figure 10a illustrates the transmission spectra of the bus MIM waveguide-coupled double D-shaped resonator structure under different ambient refractive indices (*n* = 1.00–1.20). Four distinct resonant absorption dips, labeled *λ*_11_, *λ*_22_*, λ*_33_ and *λ*_44_, are clearly resolved in Figure 10a, which corresponds to different orders of SPPs modes excited within the system. As the refractive index *n* of the medium increases progressively from 1.00 to 1.20, all Fano resonance dips exhibit a pronounced red shift. This red-shift phenomenon can be attributed to the increase in the effective refractive index of the resonator, which modifies the accumulated phase of light propagating back and forth within the cavity, thereby satisfying the resonance condition for longer wavelengths (i.e., the resonant condition *mλ* = *n*_eff_ × *L*). Concurrently, the depth and linewidth of the resonant dips also exhibit slight variations with changing refractive index, reflecting the modulation of the cavity–waveguide coupling efficiency by the ambient medium.

To further quantify the sensing performance of the proposed structure, Figure 10b presents the linear fitting results of the four resonance wavelengths as functions of the refractive index. Within the range of *n* = 1.00–1.20, *λ*_11_, *λ*_22_*, λ*_33_ and *λ*_44_ all maintain excellent linear relationships with the refractive index, with coefficients of determination (*R*^2^) all approaching unity. This indicates that the device possesses outstanding linear response characteristics, which facilitates the signal demodulation and calibration procedures in sensor systems. Based on the slopes of the linear fits, the refractive index sensitivities of the respective modes are obtained as: *S_λ_*_11_ = 1150 nm/RIU, *S_λ_*_22_ = 890 nm/RIU, *S_λ_*_33_ = 560 nm/RIU and *S_λ_*_44_ = 550 nm/RIU. These results demonstrate that the double D-shaped resonator structure not only offers the potential for multi-channel simultaneous detection, but its higher-order modes also exhibit exceptionally high sensitivity, fully proving the structure’s promising application prospects in the field of high-performance integrated photonic sensors.

Although the present study relies on numerical simulations, the practical realization of the proposed plasmonic structure is highly feasible using current nanomanufacturing technologies. The 50 nm critical dimensions of the MIM waveguide and D-shaped resonant cavities can be achieved by first depositing a high-quality silver film on a smooth substrate via magnetron sputtering, followed by precise patterning using electron beam lithography (EBL) or focused ion beam (FIB) milling. Furthermore, practical concerns regarding intrinsic metal losses have been naturally addressed in our finite element method computations, as the modified Debye dispersion model precisely captures the ohmic losses of the silver layers. In physical fabrication, slight dimensional deviations are inevitable; however, our parametric analysis demonstrates the robust fabrication tolerance of the proposed device. For instance, modifying the waveguide-resonator gap parameter (*d*) primarily causes a system shift due to waveguide perturbation. This variation alters the coupling efficiency but does not destroy the characteristic Fano resonance profiles. Similarly, minor deviations in the cavity radius (*r*) merely shift the resonance wavelengths while maintaining the sensor’s overall detection integrity. Consequently, the proposed design exhibits strong structural stability and functional reliability against potential manufacturing errors.

## 4. Conclusions

In this work, a bus MIM waveguide coupled with a D-shaped resonant cavity structure was proposed, and the transmission properties were numerically simulated using the FEM. A multi-Fano resonance phenomenon is observed in its transmission spectrum. By analyzing the influence of different structural parameters on the transmission spectra and the steady-state magnetic field distributions excited at different Fano resonance dip wavelengths, we investigate the generation mechanisms of these various Fano resonances. Results indicate that the multi-Fano resonances arise from the coupling of different resonance modes in the D-shaped resonant cavity, and the Fano resonance can be affected by the regulators of these parameters. Furthermore, we investigate refractive index sensing based on the Fano resonance by altering the refractive index of the insulator, achieving a maximum sensitivity of 1130 nm/RIU and a high figure of merit (*FOM*) of 171.49 at the first Fano resonance dip. In addition, we systematically investigated the transmission spectra and normalized magnetic field distributions of a bus MIM waveguide coupled with a double D-shaped resonator structure. By analyzing the influence of the radius of the newly added D-shaped resonant cavity on the transmission spectra and examining the normalized magnetic field distribution at the Fano resonance wavelength, we elucidated the mode mechanism underlying the generation of Fano resonance in this structure. Furthermore, by varying the refractive index of the dielectric medium in both the double D-shaped resonators and the straight waveguide, we studied the sensitivity of the structure to changes in the dielectric refractive index. The results indicate that the maximum sensitivity achieved by the double D-shaped resonator structure is 1150 nm/RIU, which represents an increase of 20 nm/RIU compared to the maximum sensitivity of the single D-shaped resonator structure. This study provides valuable insights for the design of new high-sensitivity sensors.

## Figures and Tables

**Table 1:** Comparison of the proposed sensor with other reported plasmonic sensors.

Reference	Resonator Structure	*S* (nm/RIU)	*FOM* (RIU^−1^)	Q Factor	Resonance Linewidth (nm)	Operating Wavelength (nm)
[20]	Square and circular shape	700	21.9	-	-	985
[21]	A nanoring	567.2	3.72	-	-	-
[23]	X-shaped cavity	814.95	115.52	-	-	-
[25]	Cross-shaped concave rectangular resonator	1150	73.8	-	-	-
[13]	Double rectangular cavities	596	-	-	-	620
[32]	Unique half-ring and half-square split	530.05, 620.24	15.41, 5.65	23.11, 6.49	30.40, 109.78	647, 730
[33]	Multiple circular split-ring resonator cavities	946.88	99.17	-	-	955
[34]	D-shaped cavity	1155	40	-	-	1185
This work	Dual D-shaped cavities	1130	171.49	172.99	6.59	1140

## Data Availability

The data presented in this study are available on request from the corresponding author. The data are not publicly available due to this data being supplied by Key Laboratory of Instrumentation Science & Dynamic Measurement (North University of China), Ministry of Education, and so cannot be made freely available.
